# Combining IVUS + OCT Data, Biomechanical Models and Machine Learning Method for Accurate Coronary Plaque Morphology Quantification and Cap Thickness and Stress/Strain Index Predictions

**DOI:** 10.3390/jfb14010041

**Published:** 2023-01-11

**Authors:** Rui Lv, Liang Wang, Akiko Maehara, Mitsuaki Matsumura, Xiaoya Guo, Habib Samady, Don P. Giddens, Jie Zheng, Gary S. Mintz, Dalin Tang

**Affiliations:** 1 School of Biological Science and Medical Engineering, Southeast University, Nanjing 210096, China; 2 The Cardiovascular Research Foundation, Columbia University, New York, NY 10019, USA; 3 School of Science, Nanjing University of Posts and Telecommunications, Nanjing 210023, China; 4 Department of Medicine, Emory University School of Medicine, Atlanta, GA 30322, USA; 5 The Wallace H. Coulter Department of Biomedical Engineering, Georgia Institute of Technology, Atlanta, GA 30332, USA; 6 Mallinckrodt Institute of Radiology, Washington University, St. Louis, MO 63110, USA; 7 Mathematical Sciences Department, Worcester Polytechnic Institute, Worcester, MA 01609, USA

**Keywords:** coronary vulnerable plaque, plaque models, fibrous cap thickness, vulnerable plaque model, plaque vulnerability prediction

## Abstract

Assessment and prediction of vulnerable plaque progression and rupture risk are of utmost importance for diagnosis, management and treatment of cardiovascular diseases and possible prevention of acute cardiovascular events such as heart attack and stroke. However, accurate assessment of plaque vulnerability assessment and prediction of its future changes require accurate plaque cap thickness, tissue component and structure quantifications and mechanical stress/strain calculations. Multi-modality intravascular ultrasound (IVUS), optical coherence tomography (OCT) and angiography image data with follow-up were acquired from ten patients to obtain accurate and reliable plaque morphology for model construction. Three-dimensional thin-slice finite element models were constructed for 228 matched IVUS + OCT slices to obtain plaque stress/strain data for analysis. Quantitative plaque cap thickness and stress/strain indices were introduced as substitute quantitative plaque vulnerability indices (PVIs) and a machine learning method (random forest) was employed to predict PVI changes with actual patient IVUS + OCT follow-up data as the gold standard. Our prediction results showed that optimal prediction accuracies for changes in cap-PVI (C-PVI), mean cap stress PVI (meanS-PVI) and mean cap strain PVI (meanSn-PVI) were 90.3% (AUC = 0.877), 85.6% (AUC = 0.867) and 83.3% (AUC = 0.809), respectively. The improvements in prediction accuracy by the best combination predictor over the best single predictor were 6.6% for C-PVI, 10.0% for mean S-PVI and 8.0% for mean Sn-PVI. Our results demonstrated the potential using multi-modality IVUS + OCT image to accurately and efficiently predict plaque cap thickness and stress/strain index changes. Combining mechanical and morphological predictors may lead to better prediction accuracies.

## 1. Introduction

Vulnerable plaque progression and rupture are closely related to cardiovascular disease which is the leading cause of death worldwide [1]. Accurate assessment of plaque cap thickness and prediction require accurate plaque tissue component and structure quantifications and mechanical stress/strain calculations. Plaque vulnerability is commonly understood as the likelihood of a plaque rupture causing drastic clinical events such as heart attack or stroke. While plaque vulnerability is a well-known concept, its quantitative measure is nearly impossible due to lack of plaque rupture and clinical data, which hinders its application in clinical scenarios. American Heart Association (AHA) classified plaques into Types I-VI based on histological data [2,3]. The AHA plaque classifications have been considered as the gold standard in the research community. However, it is of qualitative nature and not convenient for quantitative vulnerability tracking and predictions. Some morphological and biomechanical plaque vulnerability indices (PVIs) have been introduced based on imaging data and biomechanical factors to overcome this limitation [4,5]. Tang et al. introduced a stress-based PVI (SPVI) using 34 in vivo magnetic resonance imaging (MRI) slices from 14 human coronary plaque samples. Their SPVI plaque vulnerability assessment had an 85% agreement rate with assessment performed by histopathological analysis [4]. Goncalves et al. introduced a vulnerability index (VI) and calculated VI values for 194 patients based on histological analysis. Their follow-up data (60 months, 45 postoperative cardiovascular events registered) showed that patients with a plaque VI in the fourth quartile compared with the first to third quartiles had significantly higher risk to suffer from a future cardiovascular event (*p* = 0.0002) [5]. Wang L et al. used intravascular ultrasound (IVUS)-based morphology PVI to assess and predict plaque vulnerability [6]. Due to MRI and IVUS image resolution limitations and difficulty in recruiting large number of patients with follow-up data, accurate and reliable plaque cap thickness measurements are still difficult to obtain in vivo and PVIs still require more effort to obtain acceptance in research community and clinical practice.

Accurate and reliable image data have been employed to visualize plaque morphology and serve as the basis for plaque cap thickness predictions. Atherosclerotic plaque progression is a long and slow process lasting some 30 to 50+ years. Rapid plaque progression could also be caused by plaque destabilization followed by thrombus formation and subsequent healing. From available patient follow-up data, plaque vessel wall thickness changes were mostly under 100 µm in a year [7]. With a 150–200 µm resolution from IVUS and 200–300 µm resolution from MRI, plaque progression and morphology changes cannot be quantified for prediction with admissible reliability and accuracy. With its superior resolution of approximately 10 µm, optical coherence tomography (OCT) is able to detect thin fibrous cap (the well-known 65 µm threshold cap thickness) of vulnerable plaques [3,8,9]. Liu et al. further demonstrated that plaques with thinner fibrous cap had higher probability to have plaque rupture and thrombosis events [10]. Reith et al. determined that compared to patients with stable angina pectoris, patients with acute coronary syndrome tended to have a smaller minimal fibrous cap thickness within lipid-rich lesions [11]. Among the morphological factors characterizing vulnerable plaques such as positive remodeling, large lipid size, and macrophages infiltration, plaque cap thickness is one of the most-watched measurable characteristics for plaque prone to rupture. Efforts combining IVUS and OCT to study vulnerable plaques have been reported, and impressive results suggest that integrating two imaging modalities could be used for more accurate cap stress/strain calculations and to better evaluate plaque progression and regression [12,13]. Therefore, it can be considered that the IVUS + OCT merged data could provide detailed plaque morphological information (especially cap thickness) which forms a reliable basis for further biomechanical analysis and cap thickness and stress/strain index change predictions. 

It has been hypothesized that mechanical forces play an important role in plaque progression and rupture [14,15,16]. From a mechanical point of view, rupture occurs when plaque stress and strain at the fibrous cap exceed its tensile strength. Therefore, precise plaque stress and strain conditions may be helpful in predicting plaque rupture and critical clinical events [17,18]. Schaar et al. defined a vulnerable plaque as a plaque with a high strain region at the surface with adjacent low strain regions [19]. In addition, Zhang et al. calculated strain from in vivo image for the assessment of vulnerable plaques [20]. With the help of different evaluation methods, stress/strain variables under different definitions were calculated to detect vulnerable plaques and assess their vulnerability [4,21,22]. Those studies suggested that plaque stress and strain are closely related to plaque behaviors and could be utilized in vulnerability predictions. 

In the field of predicting plaque behaviors, most of the references adopt mixed-effect logistic regression models or Cox regression models (proportional risk regression model) [23,24]. Recently, machine learning approaches were employed in plaque progression prediction studies due to their strong predictive power and time efficiency [25]. A risk stratification model based on machine learning was used to predict all-cause death, recurrent acute myocardial infarction, and massive hemorrhage after acute coronary syndrome [26]. Lin et al. developed and validated a deep learning algorithm based on face photos to evaluate the relationship between face features and CAD [27]. By classifying the behavior of plaques, Lv et al. successfully performed a binary prediction of plaque progression based on the generalized linear mixed model and the least squares support vector machine [28]. 

Plaque vulnerability quantification and predictions have several challenges: (a) lack of rupture and clinical data to establish the gold standard for assessment and prediction; (b) selection of proper predictors, vulnerability measurements and indices to perform predictions; (c) proper biomechanical models with acceptable labor cost for potential clinical implementations. With considerable effort, we have obtained multi-modality images from ten patients with follow-up scan. The data set for each patient include IVUS + OCT + Angiography data at both baseline and follow-up. IVUS + OCT (IO) data provide us with reliable and accurate plaque morphologies which is the basis for modeling and prediction effort. Without plaque rupture and clinical events to serve as the gold standard, a cap thickness-based plaque vulnerability index (C-PVI) was introduced using IO data to serve as an alternative gold standard in this paper. While this is not the best “gold standard”, it is measurable in vivo with OCT accuracy and has the potential to be implemented in clinical practice. Several stress- and strain-based PVIs were introduced and their prediction results were compared. Time-saving 3D thin-slice models were constructed to obtain plaque stress/strain values. Values of nine morphological and biomechanical risk factors (list to be provided later) were extracted from IO images and computational models and used for prediction analysis. The random forest (RF) was adopted in this paper to predict the binary outcomes of PVI changes from baseline to follow-up. Results were compared and analyzed to identify the best single and combination predictors and the best performing PVI(s).

## 2. Materials and Methods

### 2.1. Data Acquisition, Segmentation, Slice Co-Registration and Merging

Existing de-identified IVUS, OCT and angiography data with follow-up from 10 patients (4F; mean age 70.4) were obtained from Cardiovascular Research Foundation (CRF), Columbia University, New York, NY, USA. Data were collected between April 2017 and November 2018 (mean follow-up time: 251 days) using protocol approved by local institutional review board following the rules of the Declaration of Helsinki of 1975, with informed consent obtained. Ten patients with stable angina pectoris were selected for analysis. Patients with acute coronary syndrome, severe calcified lesion, chronic total occlusion, or chronic kidney disease (Cr > 1.5 mg/dL) were excluded. Patient demographic data are shown in Table 1. Patient’s arm blood pressure was collected and used as pressure conditions in computational models. OCT images of coronary arteries were acquired using commercially available ILUMIEN OPTIS System (St. Jude Medical, Westford, MA, USA). IVUS imaging was performed using OptiCross System (OptiCross, Boston Scientific Corporation, Natick, MA, USA) with a 40 MHz IVUS catheter motorized pullback at 0.5 mm/s. Coronary angiography data were obtained for both baseline and follow-up. 

**Figure 1:**
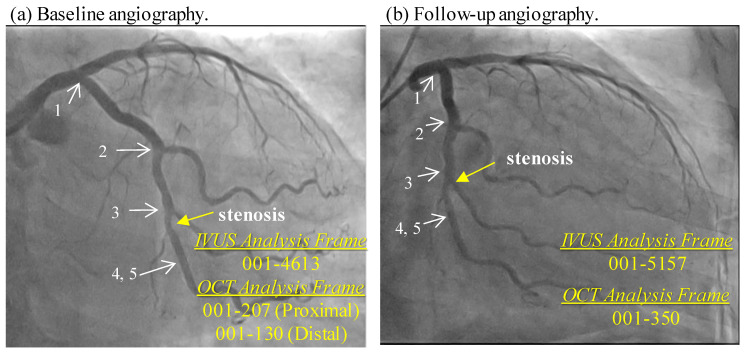
Registration of baseline and follow-up vessel segment using landmarks and vessel features (bifurcation, stenosis, and plaque components from IVUS/OCT). Only angiography is shown. (**a**) Baseline angiography; (**b**) Follow-up angiography. 1: left circumflex artery ostium, 2: 1st obtuse marginal branch, 3: 2nd obtuse marginal branch, 4: IVUS analysis start point, 5: OCT analysis start point.

**Figure 2:**
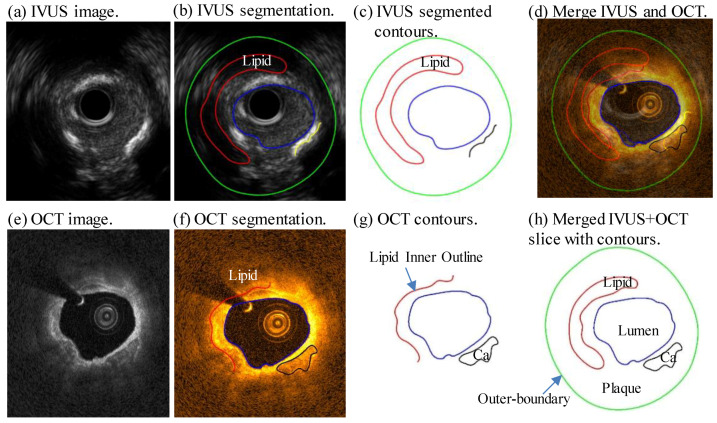
Merging OCT and IVUS contours to generate combined IVUS + OCT slice with contours. (**a**) IVUS images; (**b**) IVUS segmentation; (**c**) IVUS segmented contours; (**d**) Merge IVUS and OCT; (**e**) OCT image; (**f**) OCT segmentation; (**g**) OCT contours; (**h**) Merged IVUS + OCT slice with contours. Red: lipid; Green: outer-boundary; Blue: lumen; Black: calcification (Ca).

Co-registration of IVUS and OCT slices was performed by using fiduciary points such as side branches, bifurcations, and calcifications with the assistance of coronary angiography images (matched IVUS and OCT, matched baseline and follow-up) [29] (see Figure 1). Then, by mapping IVUS and OCT frames to the same coronary vessel segment, IVUS and OCT segmented slices were merged together to form IO slices to extract geometric contours for model construction and analysis (See Figure 2). OCT provided accurate information for plaque cap, lumen and calcification region. Plaque components included: (1) fibrous tissue (homogeneous, high backscattering region); (2) lipid-rich core (low-signal region with diffuse border) and (3) calcification (low backscattering region with sharp border). For large lipid components with a thin fibrous cap, OCT can “see” the cap clearly, but may not detect the lipid border far away from lumen due to its limited penetration. In that case, IVUS was used to obtain the lipid out-border and vessel outer-boundary. Segmentation was performed by ImageJ 1.52v software. One hundred and fourteen matched IO slices at baseline and follow-up (228 IO slices in total) were obtained from the ten patients to quantify plaque morphology and track cap thickness changes. More details about extracting plaque morphological and mechanical stress and strain data are given in Section 2.2. Sample slices with segmented contours for IVUS and OCT images were provided as supplemental material.

### 2.2. Thin-Slice Models, Morphological and Biomechanical Predictors, Data Extraction for Analysis

Three-dimensional (3D) thin-slice models were used to obtain plaque stress and strain data which will be used in plaque vulnerability predictions. A thickness of 0.5 mm was added to each IO slice to reconstruct the plaque geometry of 3D thin-slice model. A total of 228 models were constructed at baseline and follow-up. Under in vivo condition, arteries were subjected to blood pressure and axially stretched. Computational 3D thin-slice models need to start from zero-load geometries with zero pressure and stress/strain conditions. Therefore, axial and circumferential shrinking was applied to in vivo IO slices to obtain their zero-load state. Axial shrinkage was assumed to be 5% for all plaques while circumferential shrinkage rate was determined for each slice to match its in vivo morphology. Details of the pre-shrink–stretch process were described in our previous studies [30,31,32]. Vessel tissue was assumed to be hyperelastic, anisotropic, nearly-incompressible, and homogeneous. Plaque components (lipid and calcification) were assumed to be hyperelastic, isotropic, and nearly-incompressible [13]. The strain energy density functions for the isotropic and anisotropic modified Mooney–Rivlin material models are given below:

(1) $$W_{iso}=c_{1}\left(I_{1}-3\right)+c_{2}\left(I_{2}-3\right)+D_{1}\left[exp\left(D_{2}\left(I_{1}-3\right)\right)-1\right],$$

(2) $$W_{aniso}=W_{iso}+\frac{K_{1}}{K_{2}}\left\{exp\left[K_{2}{\left(I_{4}-1\right)}^{2}\right]-1\right\},$$

where$\;I_{1}=\sum \left(C_{ii}\right)$, $I_{2}=\frac{1}{2}\left[I_{1}^{2}-C_{ij}C_{ij}\right]$,$\;I_{1}$ and $I_{2}$ are the first and second invariants of right Cauchy–Green deformation tensor $C=\left[C_{ij}\right]=X^{T}X$,$\;X=\left[X_{ij}\right]=\left[\partial x_{i}/\partial a_{j}\right]$, $\left(x_{i}\right)$ is current position, $\left(a_{j}\right)$ is original position, $I_{4}=C_{ij}{\left(n_{c}\right)}_{i}{\left(n_{c}\right)}_{j}$, $n_{c}$ is the unit vector in the circumferential direction of the vessel, $c_{1}$,$\;c_{2}$, $D_{1}$, $D_{2}$, $K_{1}$ and $K_{2}$ are material parameters. Material constants of isotropic Mooney–Rivlin model from the existing literature were used: Lipid: $c_{1}$ = 0.5 kPa, $c_{2}$ = 0 kPa, $D_{1}$ = 0.5 kPa, $D_{2}$ = 1.5; Calcification: $c_{1}$ = 92 kPa,$\;c_{2}$ = 0 kPa, $D_{1}$ = 36 kPa and $D_{2}$ = 2.0; Vessel/Fibrous tissue: $c_{1}$ = −262.6 kPa, $c_{2}$ = 22.9 kPa,$\;D_{1}$ = 125.9 kPa, $D_{2}$ = 2.0, $K_{1}$ = 7.19 kPa, $K_{2}$ = 23.5 [13]. The 3D thin-slice models were solved by a finite element software ADINA 9.0 (Adina R&D, Watertown, MA, USA) to obtain plaque stress/strain distributions following our established procedures [31]. Nonlinear incremental iterative procedures were used to solve the models. Mesh analysis was performed by refining mesh density by 10% until changes in solutions became less than 2%.

**Figure 3:**
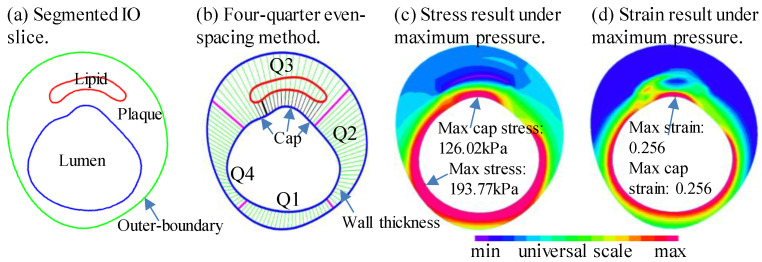
Illustration of slice contours, sample slices with Four-Quarter Even-Spacing method showing the definition and extraction of morphological and mechanical predictor data. (**a**) Segmented IO slice; (**b**) Four-Quarter Even-Spacing method. Bold black line shows the minimum cap thickness; (**c**) Stress result under maximum pressure; (**d**) Strain result under maximum pressure.

Nine morphological and mechanical risk factors were selected as predictors to predict plaque vulnerability changes from baseline to follow-up: lumen area (LA), plaque area (PA), plaque burden (PB), minimum cap thickness (MinCapT), mean cap thickness (MeanCapT), maximum cap stress (MaxCapS), mean cap stress (MeanCapS), maximum cap strain (MaxCapSn), and mean cap strain (MeanCapSn). PB was defined as

(3) $$\mathrm{PB}=\frac{\mathrm{PA}}{\mathrm{PA}+\mathrm{LA}},$$

where PA is the area between the outer boundary contour and lumen contour and LA is the area inside lumen contour (see Figure 3). Values of the risk factors were extracted for each slice using a Four-Quarter Even-Spacing method [31,32]. For each matched slice, 100 evenly spaced points from the lumen were selected and morphological and biomechanical factors from the IO slice and 3D thin-slice model at each point were obtained for analysis. Figure 3b shows a simple illustration of the method. Data extraction of the nine risk factors and following statistical analysis were implemented by MATLAB (MATLAB R2018a, MathWorks, Natick, MA, USA). 

### 2.3. Plaque Vulnerability Indices

#### 2.3.1. Cap Thickness Plaque Vulnerability Index (C-PVI)

Wang L et al. introduced 3 morphology-based indices (cap index, lipid index, and morphological index) and predicted their changes using patient IVUS follow-up data [6]. IO data can provide accurate cap thickness data, but there is no reliable information on lipid size. Bearing those in mind, and with the assumption that plaque rupture may be linked most closely to minimum cap thickness, C-PVI was introduced using MinCapT as a quantitative measure for plaque vulnerability with values 1, 2, 3 and 4 (see Table 2). Category 4 (C-PVI = 4) has the thinnest fibrous cap thickness, while Category 1 (C-PVI = 1) has the thickest fibrous cap thickness. Slice distributions for the 4 C-PVI values are given in Table 2. The cap thickness interval for each C-PVI value was chosen so that each category had some samples [33]. Some explanation is given in Section 4.1.

#### 2.3.2. Stress Plaque Vulnerability Index (S-PVI)

It is believed that plaque cap stress is closely related to plaque progression rupture and could be used as another measurement for plaque vulnerability. Two stress-based plaque vulnerability indices (S-PVI) were introduced in this paper for our quantitative vulnerability analysis: one is based on MaxCapS denoted by MaxS-PVI, one is based on MeanCapS denoted by MeanS-PVI. Stress intervals for each index values are given in Table 2. Stress interval divisions were determined so that these index values had the best match rate with C-PVI. MaxS-PVI was introduced since plaque rupture is closely linked to MaxCapS. MeanS-PVI is also considered since MeanCapS is an averaged stress value and may provide plaque stress information in a more collective way. Both stress-based indices were used for plaque vulnerability investigation in this paper to compare which one will provide better prediction results.

#### 2.3.3. Strain Plaque Vulnerability Index (Sn-PVI)

While most researchers concentrated on plaque stress (more focused on cap stress) for vulnerable plaque investigations, plaque cap strain measures whether plaque cap is stretched hard and may be a better indicator for cap mechanical conditions and plaque vulnerability. Similar to stress indices, two strain-based plaque vulnerability indices (Sn-PVI) were introduced in this paper for analysis: one is based on MaxCapSn denoted by MaxSn-PVI, one is based on MeanCapSn denoted by MeanSn-PVI. Strain intervals for each index values are given in Table 2. All arrangements for strain indices were similar to those for stress indices and are omitted for simplicity. 

#### 2.3.4. Prediction Methods and Plaque Vulnerability Predictions

All possible combinations (511 combinations) of the nine risk factors (see Section 2.2) with their values at baseline were used to predict 5 PVI changes. PVI changes were measured by changes in PVI between baseline and follow-up. Using C-PVI as an example for illustration, C-PVI change (ΔC-PVI) between baseline and follow-up for a given IO slice was defined as

(4) ΔC-PVI(Slice #)=(C-PVI at follow-up)−(C-PVI at baseline).

For simplicity, its binary outcomes BΔC-PVI (defined below) served as the target variable in our prediction models:

(5) $$B\Delta C\text{-}\mathrm{PVI}\left(\mathrm{Slice}\;\#\right)=\{\begin{array}{c} 1,\;\mathrm{if}\;\Delta C\text{-}\mathrm{PVI}>0;\\ -1,\;\mathrm{if}\;\Delta C\text{-}\mathrm{PVI}\leq 0. \end{array}$$

**Figure 4:**
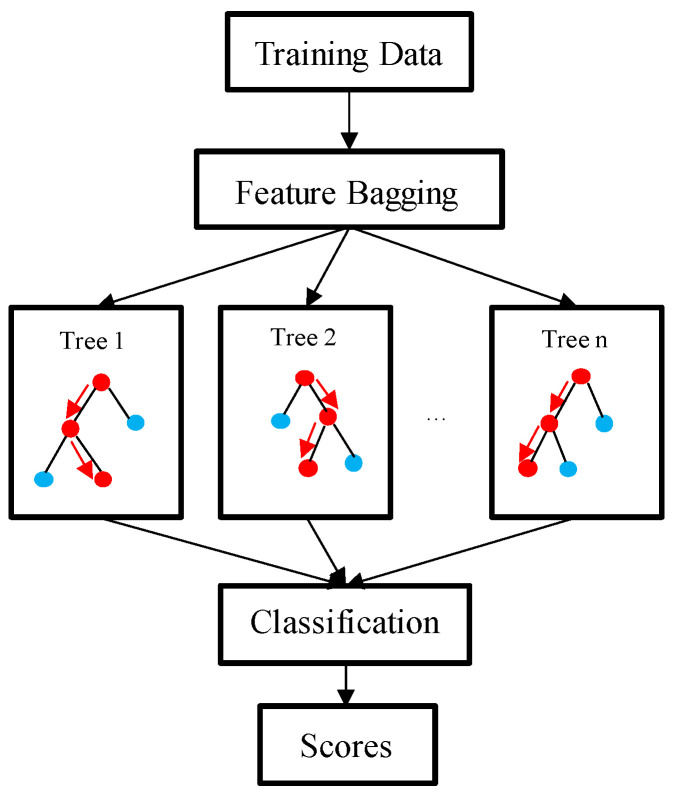
The schematic diagram of random forest model.

The same definition was used for other 4 PVIs. For each PVI, the values of the 9 risk factors at baseline and the PVI change binary outcomes of 114 slices were stored in a 10 × 114 matrix which was used as input file for the prediction methods. The RF was adopted in this paper to perform prediction. Figure 4 shows the schematic diagram of RF model. In each test, 9 risk factors and the PVI change binary outcomes from 114 slices were fed to the RF method. A standard five-fold cross-validation procedure was performed for model fitting and testing [31]. To be more specific, the data set (114 slices with baseline and follow-up scans) was randomly divided into five equal parts, with four parts used as the training set and the remaining one part used as the validation set. Then, the RF method was run five times so that each of the five parts had a chance to serve as the validation set. This five-fold cross-validation procedure was repeated 100 times (each time with new randomly divided 5 parts), and the results were averaged to obtain stable and accurate prediction results. The RF method was implemented by calling TreeBagger function in MATLAB (v2018 The MathWorks Inc., Natick, MA, USA). The number of trees in random forest was set to 50 since the prediction results (sensitivity and specificity) become stable, and further increase in the number (doubling) showed little difference in results. The procedure was repeated 100 times and the results were averaged to stabilize the prediction results. The output of the prediction was a True or False value (defined as True = ΔPVI > 0 and False = ΔPVI ≤ 0) corresponding to the optimal cutoff threshold probability) for each slice of the test set. The prediction results were compared with actual measurements of PVI changes based on IO image data (gold standard) to calculate prediction accuracy (Acc), sensitivity (Sen), and specificity (Spe) defined as follows:

(6) $$\mathrm{Acc}=\frac{\mathrm{TP}+\mathrm{TN}}{\mathrm{TP}+\mathrm{FP}+\mathrm{TN}+\mathrm{FN}},$$

(7) $$\mathrm{Sen}=\frac{\mathrm{TP}}{\mathrm{TP}+\mathrm{FN}},$$

(8) $$\mathrm{Spe}=\frac{\mathrm{TN}}{\mathrm{TN}+\mathrm{FP}},$$

where TP is the number of true positive outcomes (ΔPVI > 0 predicted as such), FP is the number of false positive outcomes (ΔPVI ≤ 0 predicted as ΔPVI > 0), TN is the number of true negative outcomes (ΔPVI ≤ 0 predicted as such), and FN is the number of false negative outcomes (ΔPVI > 0 predicted as ΔPVI ≤ 0). The abscissa of the receiver operating characteristic (ROC) curve is “1-Specificity” and the ordinate is Sensitivity. The area under ROC curve is the value of AUC. Five PVIs (C-PVI, 2 S-PVIs, and 2 Sn-PVIs) were used to identify which one would have better prediction accuracies. Details of the prediction methods and procedures were published before and are omitted here [34].

## 3. Results

### 3.1. Prediction Results for the 5 PVIs Using Combination Predictors

**Figure 5:**
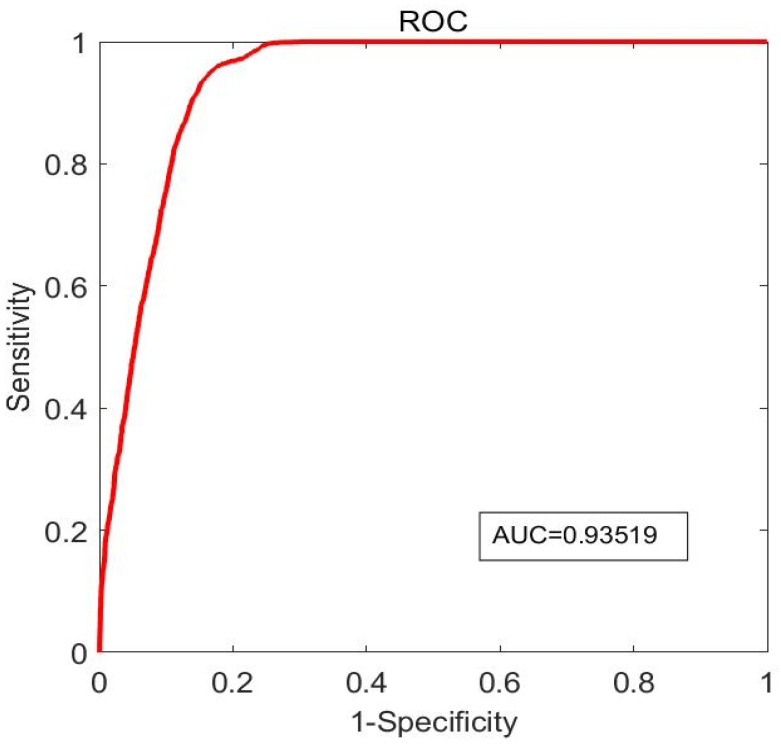
ROC curve with AUC = 0.935 for prediction of ΔMaxSn-PVI.

Table 3 lists the prediction accuracy, sensitivity, specificity, and AUC of the respective optimal combination predictors for the five PVIs. Among the five PVIs, C-PVI had the best prediction accuracy (90.3%) with the optimal predictor as a combination of PA + PB + MinCapT + MeanCapT + MeanCapSn. It also had the best specificity (95.8%). However, its sensitivity was only 56.7%. MaxSn-PVI had the best AUC (0.935) and the best Sensitivity + Specificity (1.745), with the optimal predictor as a combination of LA + PA + MaxCapSn. Its ROC curve is shown by Figure 5.

### 3.2. Prediction Results for the 5 PVIs Using Single Predictors

Prediction results of the nine single predictors for the five PVIs are shown in Table 4. Only one single predictor was used in each prediction here. Among the five PVIs, MaxSn-PVI had the best performance with MaxCapSn delivering best prediction AUC (0.909) and accuracy (79.8%). Among all nine predictors for C-PVI, PB had the best pre-diction accuracy (83.7%) and AUC (0.827). The two stress-based PVIs had lower pre-diction accuracies and AUC values. The best single predictor for MaxS-PVI was MeanCapT with accuracy of 65.4% and AUC 0.675. The best single predictor for MeanS-PVI was PA with accuracy of 75.6% and AUC 0.781. 

### 3.3. Combination Predictors Had Better Prediction Accuracies Than Those from Single Predictors

**Figure 6:**
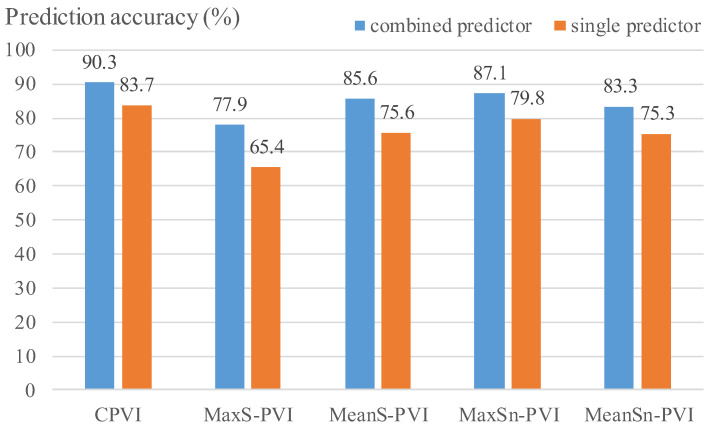
Comparison of prediction accuracies by best combination and single predictors for the five PVIs. Best combination predictors are provided in Table 3, and best single predictors are PB, MeanCapT, PA, MaxCapSn and MaxCapSn, respectively.

**Figure 7:**
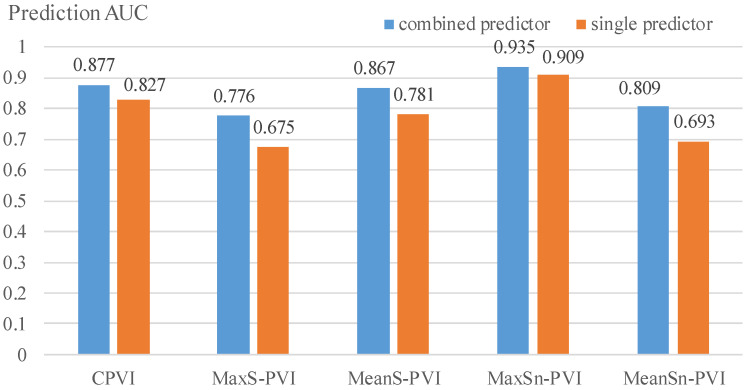
Comparison of AUC values by best combination and single predictors for the five PVIs. Best combination predictors are provided in Table 3, and best single predictors are PB, MeanCapT, PA, MaxCapSn and MaxCapSn, respectively.

Figure 6 presents prediction accuracies of the best combination predictors and the best single predictors for the five PVIs. For C-PVI, the best combination predictor increased accuracy by 6.6% compared to the best single predictor (90.3% vs. 83.7%). For MaxS-PVI, the best combination predictor had a prediction accuracy which was 12.5% over that of the best single predictor (77.9% vs. 65.4%). For MeanS-PVI, the improvement of accuracy by the best combination predictor over the best single predictor was 10.0% (85.6% vs. 75.6%). Considering MaxSn-PVI and MeanSn-PVI, the best combination predictors improved predictor accuracies by 7.3% and 8.0% over those from the best single predictors, respectively (87.1% vs. 79.8%, and 83.3% vs. 75.3%). AUC values by the best combination predictors also improved over the best single predictors by 0.050, 0.101, 0.086, 0.026, and 0.116 for the five PVIs, respectively (see Figure 7). Overall, it was observed that prediction accuracies of the best combination predictors were higher than those from the best single predictors. 

## 4. Discussion

As important as vulnerable plaque research is to the health of the general public, progress has been limited by several key factors: (a) lack of quantitative measure of plaque vulnerability; without quantitative measure, it is hard to say whether plaque vulnerability is improving or not and it is difficult to perform prediction analysis; (b) lack of accurate medical images with acceptable resolution to provide exact plaque morphology for assessment and mechanical model construction; (c) lack of “gold standard” for plaque rupture or clinical events to validate plaque progression and vulnerability predictions. In the following, we will attempt to discuss the ways in which we tried to address those limitations in this paper.

### 4.1. Introducing Quantitative Plaque Vulnerability Indices for Vulnerability Predictions

Our research effort has been focused on introducing morphological and mechanical indices for plaque classification, comparison, and prediction [4,6]. Five PVIs were introduced in this paper as measures of plaque vulnerability. With the high resolution from OCT, C-PVI was considered the “gold standard”. Our criterion for an index was that it should be based on reliable data and it should be measurable. While C-PVI may be missing some other important factors such as cell activities on the lumen surface (inflammation, erosion), it focuses on cap thickness which may be one of the most watched items for vulnerable plaques. Stress and strain indices were included because we do believe that mechanical forces play an important role in plaque progression and rupture and monitoring them may provide useful information which is not included in plaque morphology alone. Our results actually demonstrated that strain-based PVIs had better prediction accuracy compared to stress-based PVIs (see Table 3).

Compared with the current literature, Mortensen et al. noted that PB may be a major predictor of cardiovascular event and mortality risk compared to coronary stenosis [35]. The prediction results using PB for C-PVI changes (Accuracy = 83.7%, see Table 4) are in good agreement with the statement, and the best combined predictive accuracy achieved 90.3%. It should be noted that due to our data limitation, the cap thickness threshold for category 4 plaque was set to 0.2 mm in Table 2 instead of the generally accepted threshold (65 µm) of vulnerable plaques. This value was chosen for two reasons: (1) It is larger than 65 µm used in other studies based on ex vivo histology data. This is reasonable since the fibrous cap thickness in in vivo data is higher than that in histological sections [36]; (2) We could have some number of slices in Category 4 when 0.2 mm was selected. If 65 µm threshold was adopted, the number of Category 4 plaques in our data set would be zero and prediction analysis would not be possible. Hence, the well-accepted cap thickness of 65 µm for highly vulnerable plaques was not adopted due to realistic in vivo data limitations compared to results based on histological data [3]. Another point to note is that if a slice does not have a lipid core, then the slice does not have a fibrous cap and is not included in the data set in this work.

In mechanics, various evidences indicated that high plaque stresses are indeed linked to plaque rupture which is more likely to occur near thin fibrous cap, so the cap position should attract more attention [4,17]. By setting fibrous cap and its shoulder as critical region, Wang L et al. clearly explained the use of morphological factors and S-PVI to predict plaque composition changes [37]. For MeanS-PVI, the accuracy of the single predictor (PA) was 75.6%, but the optimal combination predictor was 85.6%, showing a significant improvement. Numerous studies have attempted to establish a solid link between plaque strain values and their vulnerability by solving circumferential strain directly from in vivo images [21]. Since in vivo images do not have zero-load state, strain values calculated using in vivo images used a difference reference frame and would be smaller than true strain values using zero-load reference frames [21,22]. Our models included a pre-shrink–stretch process and stress/strain were calculated using plaque zero-load geometries. Caution should be exercised when comparing results from models with different model assumptions.

### 4.2. Predicting PVI Changes Based on Accurate and Reliable OCT-Based Data

It has been mentioned that IVUS resolution is not enough to quantify thin plaque cap thickness and plaque progression, meanwhile the thin cap thickness and plaque wall thickness changes in follow-up are normally smaller than the IVUS resolution. Those limitations are the reason behind IVUS-based vulnerable plaque progression and vulnerability prediction results possibly being subjected to large errors. Using IVUS data and generalized linear mixed model (GLMM) prediction method, Wang L et al. reported that an optimal combination predictor achieved AUC = 0.629 in predicting wall thickness increase and AUC = 0.845 in predicting plaque area increase [37]. Multi-modality data combining IVUS and OCT ensures accurate cap thickness quantification, C-PVI assessment and further mechanical stress/strain calculations. These improvements made accurate and reliable plaque morphology assessment and PVI predictions possible. In this work, using IO data, the mean AUC of five PVIs is 0.853, showing a superior prediction ability. Guo et al. also reported similar findings using least squares support vector machine (SVM) prediction methods that the ability of IO/OCT-based vulnerability predictions were improved compared with IVUS-based predictions (Accuracy: 0.838 vs. 0.786) [13]. Moreover, by observing different PVIs, the accuracy and AUC value of prediction results from IO slices are both higher than 75% (see Table 3). The accuracy of all the single predictors was more than 70% for C-PVI, while PB and LA had higher prediction accuracies (86.5% and 77.8%), which confirms the general reliability of the prediction based on IO data. It is worth recognizing that fusion OCT and IVUS were used in this study to provide precise measurements of fibrous cap thickness. This data set warrants more accurate mechanical and plaque vulnerability quantification and prediction accuracy. However, it is difficult to have patients to agree to simultaneously undergo OCT, IVUS, and coronary angiography at baseline and follow-up, and thus limiting the patient data set here. For prediction methods, Wang L et al. compared various prediction methods, among which the prediction accuracy of RF was the highest, superior to SVM and GLMM. The prediction accuracy of machine learning method (RF) is 5.91% higher than that of GLMM method [34]. In this paper, the above three prediction methods were all performed and compared. The prediction results from RF were selected for report since they provided best prediction accuracies. 

### 4.3. Combining Mechanical and Morphological Predictors May Lead to Better Predictions

At present, some general scoring rules, such as CLIMA score and Burgmaier score, are often used in plaque assessment [19,38,39]. However, these scoring rules did not include mechanical forces in their assessment. It has been conjectured that those mechanical forces play an important role in plaque progression and rupture process and combining mechanical and morphological factors may lead to better prediction results. In a study using IVUS follow-up data from none patients, Wang L et al. reported that the prediction accuracy from the best morphological + mechanical combination predictor was 68.1%, 3.9% higher than that of the best morphological combination predictor (64.2%) [33]. In this paper, by using accurate multi-modality IVUS + OCT data with follow-up, for five PVIs, the combination predictors improved the prediction accuracies by 6.6%, 12.5%, 10.0%, 7.3% and 8.0% respectively, with an average improvement of 8.9%. Taking the MeanS-PVI as an example, the accuracy of the combination predictor (PA + MeanCapS) improved over that of the best single factor (PA) by 10%. That is better that the 4.01% improvement reported in work by Wang Q et al. [31]. Prediction sensitivity and specificity also had significant improvements. Our work is adding further evidence to the conjecture that combining mechanical and morphological factors may lead to better prediction results. It should be acknowledged that our sample size is small and further effort using large scale samples is needed to reach solid conclusions.

### 4.4. Labor Cost and Potential Implementations

The construction and simulation of a 3D thin-layered (slice) model could be finished within 10 min on a personal computer (Xeon E5-1620 v3 kernel processor (3.5 GHz)). This provides the possibility to integrate modeling with medical equipment for potential clinical implementations. Compared with 3D fluid-structure interaction (FSI) model (which requires approximately 2 weeks to construct one model), this 3D thin-layered (slice) model has the advantages of low labor cost, short construction time, ease of convergence, high accurate simulation results (relative error < 10% compared with FSI model). 

It is of interest to note that only baseline data would be needed to make predictions after the model becomes well trained and validated. Indeed, follow-up data (IVUS + OCT) were only needed in this paper for verification, i.e., to verify if the predictions were indeed true. It is also needed for model training as well. If data set was large enough and the model was sufficiently trained and validated, then only baseline data would be needed to make predictions. As technology develops, OCT may have better penetration and OCT alone would be enough to construct the models and provide all values for all the predictors to make predictions. 

### 4.5. Limitations

Some limitations of our study include the following. (a) Our sample size is still small and contained fewer slices with C-PVI = 3 and 4 (12.2% of total slices). That might account for the low sensitivity in our prediction results. Most of the patients were not in the acute progression stage of the disease, and the growth of plaque was generally slow, resulting in a small change in the condition of plaque within a year and a skewness distribution of data. Because most people were on medication, that also exacerbated to an unbalanced sample set. (b) Improving sample size and conducting our work at patient-level may help address sample imbalance, but lack of in vivo data on plaque rupture remains one of the limitations of current research. (c) Patient-specific vessel material properties were not available. Therefore, vessel material parameters from available literature were used in this study [30]. It should be noted that there is high variability of constitutive parameters among different individuals [40,41,42], which indeed impacts the stress/strain calculation. Guo et al. used patient-specific plaque material properties and showed that the relative errors could be 40% in stress and 123% in strain calculations if material properties obtained from ex vivo tensile testing were used [43]. (d) Thin-slice models were used in this study since they could provide better accuracy over 2D models and save model construction time compared to full 3D models [30,31]. However, they only provided plaque structure stress and strain values and did not retain flow information (for example, flow wall shear stress). Thin-slice models require much less labor to construct and could be more practical for potential clinical implementations. However, it remains true that full 3D FSI models could be a better choice for more accurate stress/strain and wall shear stress calculations. It is worth noting that the 3D thin-slice model and prediction method used in this paper are relatively more integrated, and efforts are being made to automate and streamline the whole process.

## 5. Conclusions

Since plaque vulnerability is hard to quantify, plaque cap thickness index and biomechanical stress/strain indices were defined as alternative quantitative plaque vulnerability indices (PVIs) to conduct predictive research. Accurate multi-modality IVUS + OCT data at both baseline and follow-up and machine learning methods were used to identify and validate the best predictors of changes in plaque vulnerability. The results showed that the accuracy of the combined predictors including mechanical factors was significantly better than that of the single predictors. Plaque cap thickness and cap stress and strain could be used as measurable and calculable evaluation indexes to predict plaque vulnerability change and provide a more complete early screening strategy for patients with vulnerable plaques. 

## Figures and Tables

**Table 1:** Patient demographic data. F: female; M: male; BP: blood pressure; HT: hypertension; DM: diabetes mellitus; HL: hyperlipidemia; FU: follow-up.

Patient ID	Age	Sex	BP (mmHg)	Diagnosis History	FU Days
P1	80	F	71–138	HT DM	304
P2	70	M	84–155	HT	273
P3	65	F	63–149	DM	220
P4	66	M	89–150	DM	290
P5	81	M	69–112	HT	182
P6	73	M	55–150	HT HL	248
P7	74	F	62–151	HT DM HL	244
P8	62	F	79–117	HL	195
P9	61	M	78–128	HT DM HL	283
P10	72	M	80–143	HT DM HL	272

**Table 2:** Cap- and Stress/Strain-based PVI definitions and slice divisions by PVI values.

PVI Index Values	1	2	3	4
C-PVI Min-CapT Range (mm)	(0.36, 2)	(0.26, 0.36]	(0.20, 0.26]	(0.0, 0.20]
Slice Distributions	79	21	7	7
MaxS-PVI Max Stress Range (kPa)	(20, 80]	(80, 101]	(101, 110]	(110, ∞)
Slice Distributions	73	22	8	11
MeanS-PVI Mean Stress Range (kPa)	(20, 70]	(70, 88]	(88, 93]	(93, ∞)
Slice Distributions	82	23	8	1
MaxSn-PVI Max Strain Range	(0.05,0.17]	(0.17,0.18]	(0.18,0.2]	(0.2, ∞)
Slice Distributions	80	15	13	6
MeanSn-PVI Mean Strain Range	(0.05,0.18]	(0.18,0.2]	(0.2,0.21]	(0.21, ∞)
Slice Distributions	95	13	3	3

**Table 3:** Combination predictor prediction accuracy, sensitivity, specificity and AUC values for the 5 PVIs considered.

PVI Index	Best Predictor	Acc	Sen	Spe	Sen + Spe	AUC
C-PVI	PA + PB + MinCapT + MeanCapT + MeanCapSn	0.903	0.567	0.958	1.525	0.877
MaxS-PVI	MinCapT + MeanCapT + MaxCapS	0.779	0.617	0.844	1.461	0.776
MeanS-PVI	PA + MeanCapS	0.856	0.730	0.888	1.617	0.867
MaxSn-PVI	LA + PA + MaxCapSn	0.871	0.876	0.869	1.745	0.935
MeanSn-PVI	PA + PB + MaxCapSn + MeanCapSn	0.833	0.568	0.876	1.444	0.809

Note: Total number of slices with ΔC-PVI > 0 = 16; Total number of slices with ΔC-PVI ≤ 0 = 98.

**Table 4:** Single predictor prediction accuracy, sensitivity, specificity and AUC values for the 5 PVIs considered.

**C-PVI**		**MaxS-PVI**	
Predictor	(Acc, Sen, Spe, AUC)	Predictor	(Acc, Sen, Spe, AUC)
LA	(0.702, 0.136, 0.794, 0.416)	LA	(0.566, 0.288, 0.679, 0.483)
PA	(0.738, 0.068, 0.847, 0.434)	PA	(0.568, 0.309, 0.674, 0.465)
PB	(0.837, 0.702, 0.859, 0.827)	PB	(0.634, 0.445, 0.711, 0.632)
MinCapT	(0.756, 0.199, 0.847, 0.674)	MinCapT	(0.506, 0.351, 0.569, 0.463)
MeanCapT	(0.696, 0.220, 0.774, 0.571)	MeanCapT	(0.654, 0.388, 0.762, 0.675)
MaxCapS	(0.715, 0.235, 0.793, 0.524)	MaxCapS	(0.600, 0.323, 0.713, 0.610)
MeanCapS	(0.789, 0.279, 0.872, 0.687)	MeanCapS	(0.451, 0.184, 0.559, 0.356)
MaxCapSn	(0.778, 0.293, 0.858, 0.672)	MaxCapSn	(0.559, 0.349, 0.644, 0.486)
MeanCapSn	(0.785, 0.177, 0.885, 0.603)	MeanCapSn	(0.587, 0.386, 0.669, 0.540)
**MeanS-PVI**		**MaxSn-PVI**	
Predictor	(Acc, Sen, Spe, AUC)	Predictor	(Acc, Sen, Spe, AUC)
LA	(0.735, 0.316, 0.841, 0.664)	LA	(0.576, 0.551, 0.587, 0.627)
PA	(0.756, 0.513, 0.818, 0.781)	PA	(0.704, 0.535, 0.776, 0.699)
PB	(0.676, 0.355, 0.757, 0.640)	PB	(0.615, 0.302, 0.749, 0.523)
MinCapT	(0.704, 0.171, 0.839, 0.509)	MinCapT	(0.498, 0.353, 0.560, 0.474)
MeanCapT	(0.564, 0.238, 0.646, 0.436)	MeanCapT	(0.521, 0.331, 0.602, 0.504)
MaxCapS	(0.646, 0.179, 0.764, 0.492)	MaxCapS	(0.641, 0.506, 0.698, 0.641)
MeanCapS	(0.580, 0.294, 0.653, 0.513)	MeanCapS	(0.606, 0.422, 0.684, 0.543)
MaxCapSn	(0.717, 0.277, 0.828, 0.660)	MaxCapSn	(0.798, 0.593, 0.885, 0.909)
MeanCapSn	(0.716, 0.403, 0.795, 0.628)	MeanCapSn	(0.623, 0.306, 0.757, 0.585)
**MeanSn-PVI**			
Predictor	(Acc, Sen, Spe, AUC)	Predictor	(Acc, Sen, Spe, AUC)
LA	(0.743, 0.047, 0.857, 0.508)	PA	(0.734, 0.200, 0.821, 0.451)
PB	(0.748, 0.361, 0.811, 0.579)	MinCapT	(0.710, 0.201, 0.793, 0.560)
MeanCapT	(0.714, 0.306, 0.781, 0.587)	MaxCapS	(0.701, 0.041, 0.808, 0.326)
MeanCapS	(0.715, 0.168, 0.805, 0.477)	MaxCapSn	(0.753, 0.266, 0.833, 0.693)
MeanCapSn	(0.659, 0.518, 0.682, 0.650)		

## Data Availability

Data are available on request. Data cannot be made publicly available for ethical or legal reasons (public availability would compromise patient privacy).
